# ABC Transporters and the Proteasome Complex Are Implicated in Susceptibility to Stevens–Johnson Syndrome and Toxic Epidermal Necrolysis across Multiple Drugs

**DOI:** 10.1371/journal.pone.0131038

**Published:** 2015-06-25

**Authors:** Paola Nicoletti, Mukesh Bansal, Celine Lefebvre, Paolo Guarnieri, Yufeng Shen, Itsik Pe’er, Andrea Califano, Aris Floratos

**Affiliations:** 1 Department of Systems Biology, Columbia University, New York, New York, United States of America; 2 INSERM U981, Institut Gustave Roussy, Villejuif, France; 3 Herbert Irving Comprehensive Cancer Center, Columbia University, New York, New York, United States of America; 4 Department of Biomedical Informatics, Columbia University, New York, New York, United States of America; 5 JP Sulzberger Columbia Genome Center, Columbia University, New York, New York, United States of America; 6 Department of Computer Science, Columbia University, New York, New York, United States of America; 7 Department of Biochemistry and Molecular Biophysics, Columbia University, New York, NY 10032, United States of America; 8 Institute for Cancer Genetics, Columbia University, New York, NY 10032, United States of America; Max-Delbrück Center for Molecular Medicine (MDC), GERMANY

## Abstract

Stevens–Johnson syndrome (SJS) and Toxic Epidermal Necrolysis (TEN) represent rare but serious adverse drug reactions (ADRs). Both are characterized by distinctive blistering lesions and significant mortality rates. While there is evidence for strong drug-specific genetic predisposition related to HLA alleles, recent genome wide association studies (GWAS) on European and Asian populations have failed to identify genetic susceptibility alleles that are common across multiple drugs. We hypothesize that this is a consequence of the low to moderate effect size of individual genetic risk factors. To test this hypothesis we developed Pointer, a new algorithm that assesses the aggregate effect of multiple low risk variants on a pathway using a gene set enrichment approach. A key advantage of our method is the capability to associate SNPs with genes by exploiting physical proximity as well as by using expression quantitative trait loci (eQTLs) that capture information about both cis- and trans-acting regulatory effects. We control for known bias-inducing aspects of enrichment based analyses, such as: 1) gene length, 2) gene set size, 3) presence of biologically related genes within the same linkage disequilibrium (LD) region, and, 4) genes shared among multiple gene sets. We applied this approach to publicly available SJS/TEN genome-wide genotype data and identified the ABC transporter and Proteasome pathways as potentially implicated in the genetic susceptibility of non-drug-specific SJS/TEN. We demonstrated that the innovative SNP-to-gene mapping phase of the method was essential in detecting the significant enrichment for those pathways. Analysis of an independent gene expression dataset provides supportive functional evidence for the involvement of Proteasome pathways in SJS/TEN cutaneous lesions. These results suggest that Pointer provides a useful framework for the integrative analysis of pharmacogenetic GWAS data, by increasing the power to detect aggregate effects of multiple low risk variants. The software is available for download at https://sourceforge.net/projects/pointergsa/.

## Introduction

Stevens–Johnson syndrome (SJS) and toxic epidermal necrolysis (TEN) are rare, life-threatening adverse drug reactions (ADRs) with a worldwide incidence of 1 to 4 cases over 1 million people per year [1]. SJS and TEN are variants of the same muco-cutaneous eruption, characterized by a rapidly developing blistering skin detachment. The extent of blistering is proportional to the increase in mortality rate, which is estimated between 10% and 30%.[1] SJS/TEN is a multi-drug induced hypersensitivity reaction, [1,2] promoted by a major histocompatibility class I–restricted drug clonal expansion of cytotoxic T lymphocytes (CTLs) [3]. Human leucocyte antigen (HLA) haplotypes have been implicated in drug-specific susceptibility, sometimes in an ethnicity-dependent manner. For instance, HLA-B*1502 and HLA-A*3101 are predictive markers for carbamazepine (CMZ)-induced SJS/TEN respectively in Asians [4,5] and Europeans [6], while HLA-B*5801 for allopurinol-induced SJS/TEN in both populations [7,8]. However, recent genome wide association studies (GWAS) on both Asian and European populations have failed to identify highly penetrant genetic risk factors associated with SJS/TEN across multiple drugs [9,10].

These negative findings suggest that if adverse response across many drugs is mediated by a common genetic mechanism, this mechanism is probably due to factors with small or moderate effect sizes. Non-drug-specific genetic susceptibility might modify the HLA related drug-specific predisposition and explain the localization of the cutaneous lesions. Unfortunately, the rarity of SJS/TEN limits the number of available samples and adversely affects the power of traditional GWASs to detect risk factors with moderate-to-low risk. Gene Set Analysis (GSA) methods attempt to work around the limitations of single locus association analysis by evaluating the aggregate effect of variants, specifically by investigating if genetic variation preferentially targets genes that are functionally related. [11,12] The underlying assumption is that although polygenic traits are due to the combined effect of multiple loci, their effect must coalesce through a smaller number of common biological processes. Following this hypothesis, GSA approaches that leverage GWAS hits are becoming increasingly popular in the study of unexplained hereditability [11,12].

However, results of GSA can suffer from several sources of bias, such as: 1) inaccurate SNP to gene mapping, 2) lack of correction for gene set size and gene and LD block length, 3) biological interdependence of genes within the same LD region, and 4) redundancies in gene set composition. [11,12] Here we present Pointer, an integrative GSA-based approach that overcomes these limitations. Pointer introduces several methodological innovations. First, it maps SNPs to genes using information both from LD structure and from expression quantitative trait loci that account for long-range regulatory effects. We demonstrate that the combination of these two sources of evidence leads to improved power to detect pathways that are enriched in genes harboring putative causal variants. Second, it uses a modified version of the Gene Set Enrichment Analysis (GSEA) algorithm [13] for calculating enrichment scores, controlling for gene set size, gene length, and for positive inflation due to long LD regions that contain biologically related genes, and utilizing a rigorous randomization process for the computation of the null distribution for the enrichment scores. Third, it eliminates spurious enrichment caused by compositional redundancy, e.g., when the gene sets of two pathways significantly overlap but only one of them is relevant for the phenotype.

We evaluated Pointer by analyzing genotyping data from a previously published SJS/TEN genome-wide association study [10]. We found the ABC transporters pathway to be significantly enriched for low risk genetic variants (FDR = 0.06). Members of the ABC transporter family have previously been implicated in hereditable skin disease and may play a role in drug metabolism and the tissue-specific localization of the ADR [14]. Two more pathways, the proteasome complex and propanoate metabolism pathways, were only marginally enriched (FDR = 0.25). However, in the case of the proteasome complex we were able to find independent functional evidence that corroborates its role in the pathogenesis of the SJS/TEN. Specifically, using published gene expression data, we found the proteasome complex to be enriched in genes differentially expressed in white cells located in the blistering active lesion when compared to peripheral white cells. We suggest that Pointer is a suitable tool to test the aggregate effect of markers in GWASs, especially in pharmacogenetic studies of adverse drug reactions where power can be hampered by relatively small sample sizes.

## Materials and Methods

### Genome-wide genotype data

We analyzed previously published GWAS results from 72 Caucasian SJS cases and 461 matched controls [10] genotyped with Illumina’s Human 1M BeadChip (see S1 File). Low quality SNPs were eliminated using standard quality control procedures (excluding markers with MAF < 0.01 and GenTrain score < 0.6). The association of SNPs to the ADR phenotype was calculated by logistic regression, using the top four principal components as covariates to account for population structure. Details of all analysis steps are available in [10].

### Expression QTL data

We used previously published liver-specific eQTL data [15] to account for genetic factors that affect drug catabolism and transport and which could contribute to SJS/TEN pathogenicity via the hapten-mediated mechanism [16], i.e., by enabling the formation of hapten-protein adducts that can elicit an immune response. The association between the adjusted liver expression levels and the genotypes was determined using the Kruskal-Wallis test and corrected for multiple-hypothesis testing. Significant associations were selected based on FDR ≤ 0.1. The original publication [15] provides the full details of the eQTL analysis.

### Pathway databases

We used the Kyoto Encyclopedia of Genes and Genomes (KEGG, release December 2010) which contains information for a variety of metabolic pathways, and the Reactome pathway database (release 36). The two pathway collections were chosen on the basis of being manually curated, thus ensuring high quality content. Data was downloaded from the Molecular Signature Database (MSigDB, version 3 of the C2-collections; www.broad.mit.edu/gsea/msigdb/msigdb_index.html).

### Gene expression data from SJS lesions

Chung et al. used the Affymetrix Human Genome U133 Plus 2.0 GeneChip to profile five samples of blister fluid white cells from subjects with multidrug SJS/TEN and six samples of peripheral blood mononuclear cells (PBMCs) [GSE13816], and reported the 200 most differentially expressed genes (DEGs). [3] We used DAVID [17] to calculate the enrichment of canonical pathways for those 200 DEGs.

### SNP to gene mapping

We used LD structure and eQTL information to map each genotyped SNP to all genes whose function or regulation that SNP (or one of its co-inherited variants) could potentially affect. First, the LD structure around each genotyped SNP was reconstructed. To that end, we utilized the HapMap CEU II SNP reference panel (release 22) which includes 3,572,677 common SNPs (minor allele frequency > 0.01, NCBI Build 36). Using the Haploview software [18], we computed the r^2^ between every genotyped SNP and all HapMap II SNPs within a 4 MB window (a large window size was chosen to capture both proximal and distant co-inhered variants). All HapMap II SNPs having an r^2^ ≥ 0.5 were grouped together to define a LD cluster for the genotyped SNP. Finally, genes were assigned to every SNP in a LD cluster based on: 1) Physical distance: a gene was assigned to a SNP if the SNP was located within 1500 bp upstream or downstream of the gene’s longest known transcript (gene transcript RefSeq annotation was downloaded from UCSC (hg18) [19] and mitochondrial genes coordinates from NCBI, RefSeq accession NC_012920.1); 2) Putative regulatory effect on liver gene expression: a gene was assigned to a SNP if the corresponding liver eQTL revealed a significant association (at FDR ≤ 0.1) of the SNP to the expression of the gene.

We define the set of all genes assigned to a genotyped SNP X by the process described above to be the “SNP gene map” of X, denoted as snp-map, and call X the representative SNP of the snp-map.

### Pathway enrichment analysis

Pointer uses a variant of the Gene Set Enrichment Analysis (GSEA) [13] to assess if a given pathway is enriched for GWAS SNPs. GSEA was originally developed for microarray analysis, to test whether genes in a set are collectively differentially expressed, even if no single gene achieves statistical significance on its own. Briefly, the input to GSEA is a set of genes S (e.g., genes in a pathway) and an ordered gene list L, where genes in L are ranked by the strength of their differential expression. GSEA determines whether the members of S are randomly distributed throughout L or primarily clustered at the top or bottom of the ordered list.

Our approach carefully corrects for known biases of GSA-based methods [11,12]. Such methods usually begin by mapping SNPs to genes and then rank genes according to the GWAS p-value of their mapped SNPs. However, the many-to-many nature of the SNP-to-gene mapping step can be a source of bias [20], as ranking is often performed by choosing the smallest p-value among all the SNPs mapped to a gene. This strategy favors longer genes which typically have more SNPs mapped to them, leading to systematic assignment of a smaller p-value to longer genes compared to shorter genes. The same problem exists for methods that use LD-structure to carry out the SNP to gene mapping: longer LD regions that contain many SNP will have an advantage over shorter LD regions. A third type of bias is caused by treating markers in high LD as independent GWAS hits [11,12]. For an LD region packed with several genes, this approach will transfer a single association signal to multiple genes and can cause an artificial positive inflation of the enrichment score for biological pathways which have several genes clustered in the same LD region, as it often happens [21]. In this case, although only one pathway gene may be associated with the trait, many genes will appear at the top of the GSEA ordered list, causing a spurious enrichment for the entire pathway.

To control for such positive inflation, we can attempt to construct the ordered list for GSEA by choosing only one gene from each LD region. The resulting list L in this case would comprise a subset of genes, unlike the original GSEA method where all genes arrayed on the gene expression microarray chip are used. A downside of this approach is that it can discriminate against pathways whose genes are under-represented in L. To avoid such discrimination, Pointer builds a separate ordered list L_P_ for each pathway P. Specifically, given the set G_P_ of genes in P, we process all snp-maps in order of increasing p-value of their representative SNP. From each snp-map we randomly choose one gene to add to the ranked list L_P_, giving preference to genes from G_P_ in order to maximize the representation of G_P_ in L_P_. After a snp-map has been processed all its remaining genes are removed from the leftover snp-maps, thus making them ineligible for inclusion in L_P_. As snp-maps are constructed to, effectively, span LD regions, this procedure guarantees that only one gene per LD region is included in L_P_. Finally, the GSEA gene set S_P_ corresponding to P is defined as the subset of G_P_ included in L_P_.

**Fig 1:**
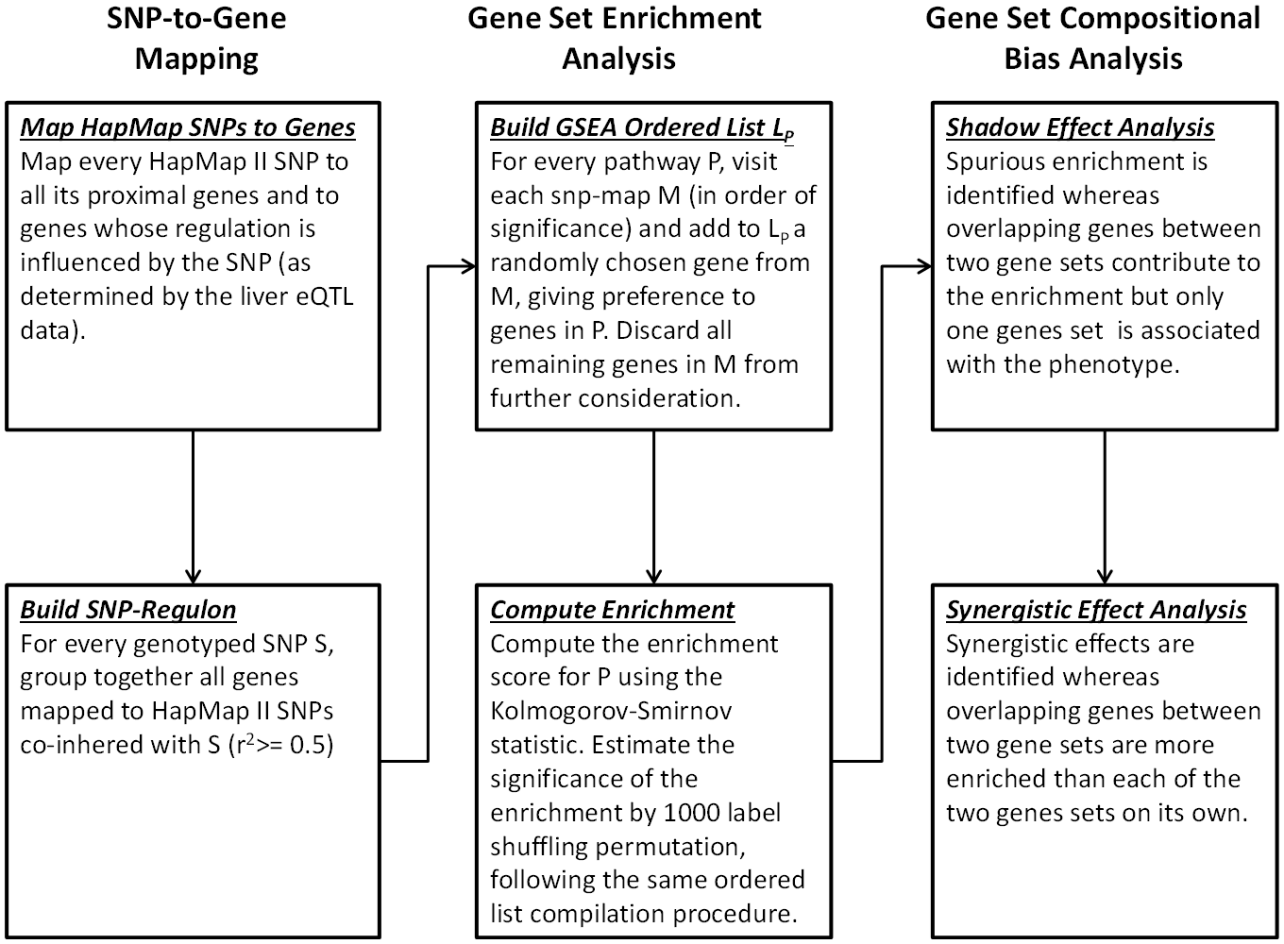
Key steps of the Pointer algorithm.

Genes in L_P_ are ordered according to the GWAS p-value of the representative SNP of their originating snp-map. The negative log of this p-value is used as the correlation metric in the standard GSEA enrichment score (ES) calculation for S_P_ [13]. A null distribution for ES is empirically estimated by repeating the process described above for 1000 case/control label shuffling permutations (S1 Fig). This permutation based procedure corrects for the length biases identified previously (gene length and LD-region length) and for the preferential selection from G_P_ when constructing L_P_. Additionally, the null distributions allow us to (1) compute a normalized enrichment score (NES) that is comparable among gene sets of different size, and (2) build a null distribution of NES that can be used to calculate a false discovery rate (FDR) for any NES threshold. An illustration of the entire process is shown in Fig 1. Procedural details of our GSEA adaptation can be found in the Supplementary Materials, S2 File.

### Shadow and synergistic pathways

Compositional biases in pathway collections may lead to overrepresentation of particular biological processes, resulting in the inclusion of multiple functionally related pathways in the pathway collection [12]. When the gene sets of two pathways overlap significantly, spurious enrichment may occur if only one is involved in a particular phenotype. In this case, the other pathway (called “shadow” pathway) can appear significantly enriched just because it shares a large number of genes with the truly enriched one. Another possibility is that two pathways are synergistically associated with a phenotype, i.e., that the intersection of the two pathways’ gene sets is more “enriched than either gene set alone.

To identify both shadow and synergistic effects we used the method described in Lefebvre et al. [22]. Specifically, we evaluated every pair of significantly enriched pathways (FDR ≤ 0.25) by testing whether (1) either of them was no longer enriched after the overlapping genes were excluded from the gene set (shadow effect), or (2) the set of their shared genes was more enriched than any of the individual pathways (synergistic effect). Details of the process are provided in the Supplementary Materials, S3 File.

## Results

### ABC transporters and Proteasome pathways are enriched in genetic risk variants for SJS/TEN

**Fig 2:**
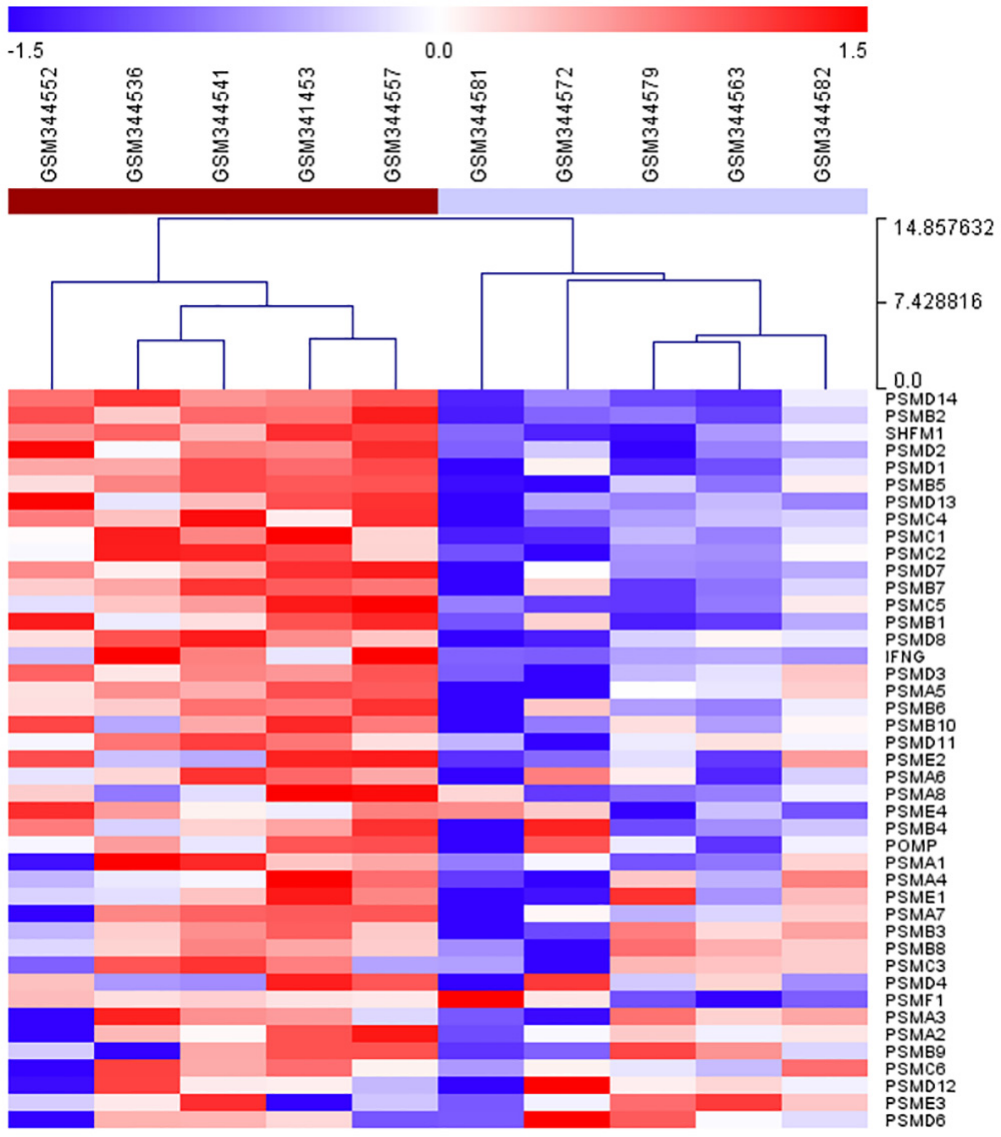
QQ-plot (panel A) and GSEA plots (panel B) of the KEGG ABC transporter pathway. The QQ-plot is constructed using the genotyped SNPs whose snp-map contains at least one ABC transporter pathway gene. The GSEA plot shows the enrichment score of the ABC transporter pathway. The top portion of the plot shows the running enrichment score for the pathway genes as the analysis moves down the ranked list. The peak score is the enrichment score for the gene set. The bottom portion of the plot shows the value of the ranking metric as it moves down the list of ranked genes. The plots for the other two enriched pathways (Proteasome and Propanoate metabolism) look similar (see S2 Fig).

837,175 SNPs passed the quality control filters applied to the genotypic data [10] and 423,718 were mapped to at least one gene by our SNP-to-gene mapping process (66,621 were assigned to two or more genes). Using the logistic regression analysis results from [10] we applied Pointer on all KEGG pathways comprising enough genes to satisfy the GSEA-recommended minimum gene set size [23]; there were 153 such pathways. After controlling for spurious enrichment (shadow analysis), we identified the ABC transporters, Proteasome and Propanoate metabolism as the top three most enriched pathways (FDR < 0.25; Table 1). The ABC transporters pathway was the top enriched (p-value = 10^−3^ and FDR < 0.06). The QQ-plots constructed for each pathway indicate that SNPs from the pathway genes are significantly more associated with SJS/TEN than expected by chance (Fig 2 and S2 Fig). The lists of SNP-to-GENEs and the associated p-value are reported in S1 Table.

**Table 1:** The top enriched KEGG pathways for low risk genetic variants ranked by normalized enrichment score (NES).

**Pathway**	#GSS	#GLE	ES	NES	PV	FDR
**ABC transporters**	44	23	0.6	3.4	0.001	0.06
**Proteasome**	43	21	0.4	2.6	0.004	0.25
**Propanoate metabolism**	32	13	0.5	2.5	0.002	0.25

Abbreviations: #GSS (gene set size for the pathway); #GLE (number of pathway genes in GSEA leading edge); ES (enrichment score), NES (normalized enrichment score), PV (p-value of ES), FDR (false discovery rate).

### The proteasome pathway is enriched in differentially expressed genes from SJS active lesions

**Fig 3:**
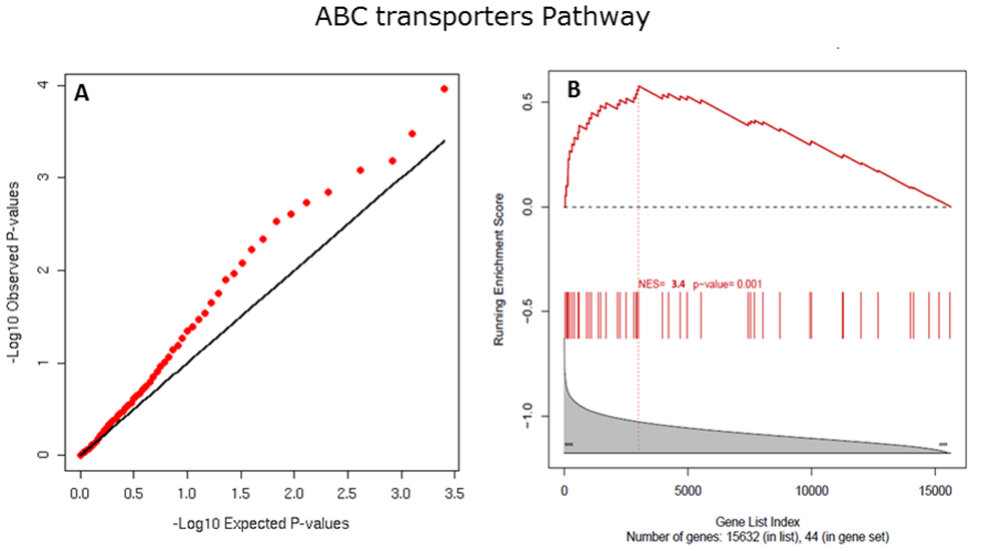
Hierarchical clustering of expression profiles for genes in the KEGG proteasome pathway. The Hierarchical clustering analysis was performed on gene expression data from Chung et al., 2008. Individual gene-related signals are increased (red), unchanged (white), or decreased (blue). The analysis clearly separates cases (brown group) from controls (light blue group) and reveals the up-regulation of proteasome genes in the SJS/TEN lesions.

We used DAVID [17] to test for KEGG pathways enriched in a previously published list of 200 differentially expressed genes (DEGs) from SJS/TEN active lesions [3]. The Proteasome pathway was identified as the most significantly enriched (FDR = 0.001, Table 2). Hierarchical clustering of the expression profiles of the KEGG proteasome genes clearly delineated case and control samples and demonstrated the up-regulation of the proteasome genes in the SJS/TEN lesions (Fig 3). The overlap between the Pointer and the DAVID enrichment results was found to be significant (Fisher’s exact text p-value of 0.03). This concordance of findings between the SNPs and expression analyses provide additional evidence for the important role of the proteasome complex in the pathogenesis of SJS/TEN.

**Table 2:** The top KEGG pathways enriched in differentially expressed genes from SJS/TEN active lesions and ranked by FDR (FDR<0.25).

**KEGG Pathways**	#GENES	PV	FE	FDR
Proteasome (*)	6	0.00001	11.4	0.001
**Lysosome**	6	0.013	4.17	0.148

The enrichment score is computed by DAVID on the 200 DEGs from Chung et al. Abbreviations: #GENES (number of DEGs in the pathway), PV (p-value, Fisher Exact test), FE (Fold Enrichment), FDR (false discovery rate). A (*) next to a pathway name indicates that the pathway was found to be enriched by both Pointer and DAVID.

### The role of the proteasome genes in SJS/TEN

The proteasome complex genes were found to be enriched in SJS/TEN risk variants and were also significantly up-regulated in the SJS/TEN skin active lesions. The complex is implicated in several well-known biological processes, many of which are reported in the Reactome database. Indeed, the database contains 24 proteasome complex-related pathways. Therefore, we applied Pointer to the Reactome collection, aiming to more precisely characterize the proteasome complex processes that are relevant for SJS/TEN susceptibility. Additionally, given that Reactome contains a different set of biological processes than the ones found in KEGG, we reasoned that Pointer might discover more enriched pathways. We note that the version of Reactome available through the MSigDB lacks pathways related to ABC transporter genes.

After shadow analysis we identified 11 significantly enriched pathways with FDR < 0.25 (Table 3). 10 of the 11 enriched pathways, including the top three, were proteasome complex-related pathways. All of them were pairwise synergistic, each sharing 38 genes with the KEGG proteasome complex. To investigate whether other genes, beyond the 38 shared genes, contributed appreciably to the enrichment of these 10 pathways we performed shadow analysis against the KEGG proteasome complex pathway. Through this analysis we aimed to identify which pathways were independently enriched due to variants in genes other than those in the KEGG proteasome complex. Three of the 10 Reactome pathways were not shadowed by the KEGG proteasome: (i) Apoptosis, (ii) cyclin E associated events during G1-S transition, and (iii) scf beta trcp mediated degradation of emi1. The apoptosis pathway was also found to be significantly enriched in differentially expressed genes from SJS/TEN active lesions (DAVID analysis, FDR = 0.05, Table 4).

**Table 3:** Enriched Reactome pathways ranked by FDR.

**Pathway**	GSS	GLE	ES	NES	PV	FDR	SD
**CYCLIN E ASSOCIATED EVENTS DURING G1 S TRANSITION**	58	30	0.40	3.49	0.001	0.08	0.12
**REGULATION OF ORNITHINE DECARBOXYLASE**	47	24	0.42	3.30	0.001	0.07	NA
**APOPTOSIS**	127	62	0.37	3.06	0.001	0.10	0.12
**P53 INDEPENDENT DNA DAMAGE RESPONSE**	43	21	0.41	2.99	0.003	0.09	NA
**Generic transcription pathway**	35	23	0.51	2.98	0.001	0.08	-
**SCF BETA TRCP MEDIATED DEGRADATION OF EMI1**	48	23	0.39	2.77	0.003	0.12	0.14
**VIF MEDIATED DEGRADATION OF APOBEC3G**	47	22	0.38	2.67	0.005	0.13	NA
**STABILIZATION OF P53**	46	21	0.38	2.63	0.005	0.13	NA
**SCF SKP2 MEDIATED DEGRADATION OF P27 P21**	52	21	0.36	2.59	0.005	0.13	0.07
**G1 S TRANSITION**	96	37	0.33	2.34	0.011	0.22	0.03
**REGULATION OF APC ACTIVATORS BETWEEN G1 S AND EARLY ANAPHASE**	71	26	0.33	2.26	0.016	0.23	0.03

In capital letters, pathways that contain KEGG proteasome genes.

Abbreviations: GSS (pathway gene set size); GLE (number of genes in leading edge); ES (Enrichment score), NES (Normalized Enrichment score), PV (p-value), FDR (false discovery rate), SD (p-value of shadow analysis against KEGG proteasome pathway)

**Table 4:** The top REACTOME pathways enriched in differentially expressed genes from SJS/TEN active lesions and ranked by FDR (FDR<0.25).

**Reactome Pathways**	#GENES	PV	FE	FDR
**DNA Replication**	11	0.0009	3.51	0.009
**Cell Cycle Checkpoints**	8	0.001	4.65	0.014
**Cdc20:Phospho-APC/C mediated degradation of Cyclin A**	6	0.003	5.91	0.029
**Immune System**	22	0.003	1.91	0.029
**Proteasome mediated degradation of PAK-2p34**	5	0.004	7.08	0.046
Apoptosis (*)	8	0.005	3.65	0.052
**Cell Cycle, Mitotic**	12	0.007	2.49	0.066
**HIV Infection**	9	0.008	3.06	0.074
**Signaling by Wnt**	5	0.01	5.31	0.121

The enrichment score is computed by DAVID on the 200 DEGs from Chung et al. Abbreviations: #GENES (number of DEGs in the pathway), PV (p-value, Fisher Exact test), FE (Fold Enrichment), FDR (false discovery rate). A (*) next to a pathway name indicates that the pathway was found to be enriched by both Pointer and DAVID.

We could not test the shadow effect for 4 of the 10 pathways because they had too few non-proteasome genes (< 10). One among these four, the Regulation of ornithine decarboxylase, was the only pathway that had a normalized enrichment score greater than that of the KEGG proteasome (3.3 and 2.6, respectively) and did not have any overlapping genes with apoptosis, cyclin E associated events during G1-S transition, and scf beta trcp mediated degradation of emi1 beyond the KEGG proteasome genes. These findings suggest that the role of the proteasome complex in the genetic susceptibility of the SJS/TEN may be a manifestation of the genetic perturbation of specific biological processes (such as Apoptosis, cyclin E associated events during G1-S transition, and scf-beta-trcp mediated degradation of emi1). Regulation of ornithine decarboxylase may be also involved as it has a higher enrichment score than the KEGG proteasome complex and its non-proteasome genes do not overlap with the other pathways.

### Impact of SNP-to-gene mapping on the power to detect pathway enrichment

The SNP-to-gene mapping step in Pointer integrates information from both local LD structure and liver eQTL data. To evaluate the benefits of this combined approach, we compared it with two mapping strategies often used in other GSA methods: 1) Physical distance method: we assigned each genotyped SNP to its closest gene by physical distance alone [24], 2) LD-reconstruction method: we followed Pointer’s snp-map building process but excluded the eQTL data [21]. In total we mapped respectively 357,097 SNPs and 416,812 SNPs to at least one gene. We repeated our pathway enrichment analysis for these two SNP-to-gene maps. In both cases, considering an FDR < 0.25, no KEGG pathway was found to be significantly enriched (Table 5). As these results suggest, increasing the information used at the SNP-to-gene mapping step (from physical distance alone, to local LD structure, to LD structure plus eQTL data) leads to improved power to detect enriched pathways, with the eQTL data seemingly having the largest incremental impact. This finding corroborates recent observations that eQTLs are highly informative in interpreting GWAS results [25].

**Table 5:** Comparison of False Discovery Rates from the pathway analysis performed with three SNP-to-gene mapping strategies.

**Pathway**	FDR^1^	FDR^2^	FDR^3^
**ABC transporters**	0.86	0.64	0.06
**Proteasome**	0.98	0.68	0.25

Abbreviations: FDR1 (FDR from the pathway analysis performed using physical distance only), FDR2 (FDR from the pathway analysis performed using LD-reconstruction), FDR3 (FDR from the pathway analysis performed with Pointer).

### Comparison with other GSA methods

We benchmarked the performance of Pointer against 2 well-cited gene set enrichment methods: Magenta [26] and i-Gsea4gwas-V2 [27]. Both software tools were executed with default parameter settings and using as input the SJS/TEN GWAS data and the KEGG pathway database. The two algorithms utilize somewhat different approaches for the key GSA computational steps (SNP-to-gene mapping and testing for enrichment). Magenta maps SNPs to genes by physical distance and, similar to Pointer, corrects for LD structure. Pathway enrichment is estimated as the overrepresentation of leading edge genes relative to a permutation-based null distribution. Magenta reported no significant pathways after correcting for multiple testing. Although not overall significant, the ABC transporter and Proteasome pathways were among the top four nominally enriched associated pathways (p-value = 0.036 –rank second, p-value = 0.057—rank fourth, respectively; all p-values uncorrected). I-Gsea4gwas-V2 carries out the SNP-to-gene mapping using only physical proximity information. It utilizes the Kolmogorov–Smirnov test for computing enrichment and uses SNP label permutation to compute the null score distribution. I-Gsea4gwas-v2 reported 15 enriched pathways at the 0.1 FDR level (S2 Table), including the ABC transporter pathway (nominal p-value = 0.004 and FDR = 0.05) but not the proteasome pathway (nominal p-value = 0.152 and FDR = 0.5).

In summary, results from Pointer, Magenta, and i-Gsea4gwas-V2 point in the same direction, albeit with varying degrees of statistical strength. The ABC transporters and Proteasome pathways consistently appear among the top scoring pathways. In the case of the Proteasome pathway, enrichment is stronger when the SNP-to-gene mapping corrects for LD and integrates eQTL data, underscoring the effect of using a more comprehensive mapping approach. The inclusion of the eQTL data in particular, in conjunction with the use of a rigorous label shuffling process for estimating the null distribution of enrichment scores, seems to confer an appreciable power gain to Pointer compared to other approaches.

## Discussion

We presented Pointer, a novel approach that integrates information from liver eQTLs and linkage disequilibrium structure with GWAS association signals to aggregate the modest risk effect of common genetic variants through canonical pathways. Applying Pointer, we identified a number of pathways enriched in low risk variants, suggesting they may play a role in SJS/TEN genetic predisposition across multiple drugs.

Pointer is a GSA-based approach that introduces several methodological innovations to address known constraints and biases of enrichment analysis, thus significantly improving statistical power. There is strong evidence that SNPs associated with complex traits are most likely acting by affecting gene regulation [25]. Motivated by this observation we used liver eQTL information to capture regulatory pharmacogenetic variants. Existing GSA approaches map SNPs to genes using either regulatory information [23,28] or physical distance [24,29]. By combining these two mapping strategies we showed that Pointer improves the power of enrichment analysis, even in studies with limited sample size. We demonstrated that our SNP-to-gene mapping method was essential in detecting enrichment (Table 5). Additionally, we carefully controlled for inflation of enrichment scores due to long LD regions that contain multiple genes from the same pathway, by selecting only one representative gene per region. Finally, we addressed compositional redundancy among gene sets by identifying spurious enrichments.

When Pointer was applied to the KEGG pathway collection it identified the ABC transporters pathway (ABC-P) as the most significantly enriched in genetic variants associated with SJS/TEN. The ABC-P is a collection of 44 genes from the same gene family. Genetic alterations in several of these genes have been linked to congenital keratinization disorders such as Pseudoxanthoma Elasticum (causal mutations in ABCC6), Harlequin ichthyosis (ABCA12) and lamellar ichthyosis (ABCA12), a form of ichthyosis characterized by skin desquamation over the whole body. [14] Notably, multidrug resistance transporters also belong to a subclass of this family [30]. This suggests that multiple risk variants distributed across several of these genes may contribute to SJS/TEN predisposition in a non-drug specific manner and may explain the localization of the ADR target tissue.

Pointer also identified the proteasome complex and the propanoate metabolism pathways as marginally enriched in low-risk variants. For the proteasome complex, independent functional evidence further corroborated the role of the pathway in the pathogenesis of SJS/TEN. Specifically, we showed that the pathway is enriched not only in genetic variants but also in genes which are differentially expressed in blister cells from skin lesions of SJS-TEN versus PBMCs [3]. As the proteasome complex participates in several biological processes that mediate protein degradation, we further investigated if any of them are specifically implicated in SJS/TEN. To that end we applied Pointer to the Reactome database which, to the best of our knowledge, has the most comprehensive collection of Proteasome-related pathways. 10 of the 24 Reactome Proteasome-related pathways were identified as enriched by Pointer. For the majority of them, enrichment was entirely due to genes shared with the KEGG proteasome complex pathway. But for 3 pathways (Apoptosis, cyclin E associated events during G1-S transition, and, scf-beta-trcp mediated degradation of emi1) variants in genes other than those in the KEGG proteasome complex were also significant contributors to the enrichment score. This suggests that these pathways may represent specific biological processes through which the proteasome complex is involved in the manifestation of SJS/TEN.

More broadly, there are at least two mechanisms that could explain the contribution of the proteasome complex in the pathogenesis of SJS/TEN. First, proteasome mediated protein degradation is a mechanism that is critically involved in generating the repertoire MHC-I presented peptides after interferon-γ activation [31,32,33]. Genetic determinants might control the activity of the immune-proteasome in response to a drug-antigen. Second, proteasome-mediated ubiquitin-dependent protein degradation is crucial for cell survival [34]. In particular, the activation of the proteasome seems to play a role in the cyclin-E dependent proliferation, activation and apoptosis of T lymphocytes and neutrophils [35,36]. Indeed, proteasome inhibitors have been shown to induce the in vitro apoptosis of natural killer cells [37] (the major effectors in the SJS immunoreaction) and to reduce T-cell dependent skin reaction in Contact Hypersensitivity reactions in mice [38]. Therefore it seems plausible that genetically-induced functional alterations in this complex and other apoptotic genes could increase the hapten-specific survival of lymphocytes as well as the severity of the immunoreaction. This hypothesis is supported by the fact that the apoptosis pathway was enriched both in risk variants as well as in differentially expressed genes. Further evidence for the genetic regulation of neutrophil survival by the proteasome complex is provided by three large genome-wide association studies which showed that neutrophil count is affected by genetic determinants in PSMD3 (proteasome 26S subunit, non-ATPase, 3) [39,40], which regulate the expression of the gene [40].

Finally, ornithine decarboxylase (ODZ) may also play a role in SJS/TEN predisposition. It has been shown that an increase in the enzyme’s activity in keratinocites suppresses a classic hapten induced immune-response in the context of Contact Hypersensitivity reactions [41]. The proteasome complex is involved in the degradation of ODZ and high expression of the complex could lead to dysregulation of the lymphocytic immune response specifically on the skin. In conclusion, we developed Pointer, an integrated pharmacogenetic GSA approach which improves the power to detect the aggregate effect of common genotyped SNPs using linkage data and liver-specific regulatory information. We applied this approach to publicly available SJS/TEN GWAS data and we found that the ABC transporters and Proteasome pathways were significantly enriched in low-risk genetic variants. Moreover, proteasome genes were highly expressed in activated lymphocytes collected from multi drugs SJS/TEN lesions. This evidence suggests a role for the proteasome complex in the pathogenesis of blistering lesions as well as in genetic predisposition to non-drug specific SJS/TEN. Further replication and functional studies are needed to confirm these hypotheses.

## Supporting Information

S1 Fig Pseudo code explains the analytic procedure used to build the enrichment score null distribution. (TIF)

S2 Fig QQ-plot (A) and GSEA-plot (B) of the KEGG Proteasome complex and Propanoete metabolism pathways. The SNPs presented in each plot are the representative SNPs for all genes in the corresponding pathway. The GSEA plots show the enrichment score of the two pathways. The top portion of each plot shows the running enrichment score for the pathway genes as the analysis moves down the ranked list. The peak score for each plot is the enrichment score for the gene set. The bottom portion of each plot shows the value of the ranking metric as it moves down the list of ranked genes.
(TIF)

S1 File GWAS data availability. (DOCX)

S2 File Empirical derivation of enrichment score null distribution and Normalized enrichment score. (DOCX)

S3 File Shadow analysis and Synergy analysis. (DOCX)

S1 Table Top associated SNPs to gene in ABC and proteasome pathway. (DOCX)

S2 Table The two tables show the pathway enrichment results from I-GSEA4GWAS v2 in panel A and from Magenta in panel B. (DOCX)

S3 Table ID samples considered in the analysis. (XLSX)

## References

[1] Roujeau JC, Stern RS (1994) Severe adverse cutaneous reactions to drugs. N Engl J Med 331: 1272–1285. 7794310 10.1056/NEJM199411103311906

[2] Roujeau JC, Guillaume JC, Fabre JP, Penso D, Fléchet ML, Girre JP (1990) Toxic epidermal necrolysis (Lyell syndrome). Incidence and drug etiology in France, 1981–1985. Arch Dermatol 126: 37–42. 2134982 10.1001/archderm.126.1.37

[3] Chung W-H, Hung S-I, Yang J-Y, Su S-C, Huang S-P, Wei C-Y, et al (2008) Granulysin is a key mediator for disseminated keratinocyte death in Stevens-Johnson syndrome and toxic epidermal necrolysis. Nat Med 14: 1343–1350. 10.1038/nm.1884 19029983

[4] Chung WH, Hung SI, Hong HS, Hsih MS, Yang LC, Ho HC (2004) Medical genetics: a marker for Stevens-Johnson syndrome. Nature 428: 486 15057820 10.1038/428486a

[5] Hung SI, Chung WH, Jee SH, Chen WC, Chang YT, Lee WR (2006) Genetic susceptibility to carbamazepine-induced cutaneous adverse drug reactions. Pharmacogenet Genomics 16: 297–306. 16538176 10.1097/01.fpc.0000199500.46842.4a

[6] McCormack M, Alfirevic A, Bourgeois S, Farrell JJ, Kasperaviciute D, Carrington M, et al (2011) HLA-A*3101 and carbamazepine-induced hypersensitivity reactions in Europeans. N Engl J Med 364: 1134–1143. 10.1056/NEJMoa1013297 21428769 PMC3113609

[7] Lonjou C, Borot N, Sekula P, Ledger N, Thomas L, Halevy S (2008) A European study of HLA-B in Stevens-Johnson syndrome and toxic epidermal necrolysis related to five high-risk drugs. Pharmacogenet Genomics 18: 99–107. 10.1097/FPC.0b013e3282f3ef9c 18192896

[8] Hung SI, Chung WH, Liou LB, Chu CC, Lin M, Huang HP (2005) HLA-B[ast]5801 allele as a genetic marker for severe cutaneous adverse reactions caused by allopurinol. Proc Natl Acad Sci USA 102: 4134–4139. 15743917 10.1073/pnas.0409500102 PMC554812

[9] Ueta M, Sotozono C, Tokunaga K, Yabe T, Kinoshita S (2007) Strong association between HLA-A[ast]0206 and Stevens-Johnson syndrome in the Japanese. Am J Ophthalmol 143: 367–368. 17258541 10.1016/j.ajo.2006.09.029

[10] Shen Y, Nicoletti P, Floratos A, Pirmohamed M, Molokhia M, Geppetti P, et al (2011) Genome-wide association study of serious blistering skin rash caused by drugs. Pharmacogenomics J. 10.1038/tpj.2010.84 21221126

[11] Wang K, Saito M, Bisikirska BC, Alvarez MJ, Lim WK, Rajbhandari P, et al (2009) Genome-wide identification of post-translational modulators of transcription factor activity in human B cells. Nat Biotech 27: 829–837. 10.1038/nbt.1563 PMC2753889 19741643

[12] Wang L, Jia P, Wolfinger RD, Chen X, Zhao Z (2011) Gene set analysis of genome-wide association studies: Methodological issues and perspectives. Genomics 98: 1–8. 10.1016/j.ygeno.2011.04.006 21565265 PMC3852939

[13] Subramanian A, Tamayo P, Mootha VK, Mukherjee S, Ebert BL, Gillette MA, et al (2005) Gene set enrichment analysis: A knowledge-based approach for interpreting genome-wide expression profiles. Proc Natl Acad Sci U S A 102: 15545–15550. 16199517 10.1073/pnas.0506580102 PMC1239896

[14] Uitto J (2005) The gene family of ABC transporters—novel mutations, new phenotypes. Trends in Molecular Medicine 11: 341–343. 15996518 10.1016/j.molmed.2005.06.004

[15] Schadt EE, Molony C, Chudin E, Hao K, Yang X, Lum PY, et al (2008) Mapping the genetic architecture of gene expression in human liver. PLoS Biol 6: e107 10.1371/journal.pbio.0060107 18462017 PMC2365981

[16] Park BK, Pirmohamed M, Kitteringham NR (1998) Role of Drug Disposition in Drug Hypersensitivity: A Chemical, Molecular, and Clinical Perspective. Chemical Research in Toxicology 11: 969–988. 9760271 10.1021/tx980058f

[17] Huang DW, Sherman BT, Lempicki RA (2008) Systematic and integrative analysis of large gene lists using DAVID bioinformatics resources. Nat Protocols 4: 44–57. 10.1038/nprot.2008.211 19131956

[18] Barrett JC, Fry B, Maller J, Daly MJ (2005) Haploview: analysis and visualization of LD and haplotype maps. Bioinformatics 21: 263–265. 15297300 10.1093/bioinformatics/bth457

[19] Fujita PA, Rhead B, Zweig AS, Hinrichs AS, Karolchik D, Cline MS, et al (2010) The UCSC Genome Browser database: update 2011. Nucleic Acids Research. 10.1093/nar/gkq963 PMC3242726 20959295

[20] Kraft P, Raychaudhuri S (2009) Complex Diseases, Complex Genes: Keeping Pathways on the Right Track. Epidemiology 20: 508–511 510 10.1097/EDE.0b013e3181a93b98 19525687 PMC2704602

[21] Hong M-G, Pawitan Y, Magnusson P, Prince J (2009) Strategies and issues in the detection of pathway enrichment in genome-wide association studies. Human Genetics 126: 289–301. 10.1007/s00439-009-0676-z 19408013 PMC2865249

[22] Lefebvre C, Rajbhandari P, Alvarez MJ, Bandaru P, Lim WK, Sato M, et al (2010) A human B-cell interactome identifies MYB and FOXM1 as master regulators of proliferation in germinal centers. Mol Syst Biol 6. 10.1038/msb.2010.31 PMC2913282 20531406

[23] Zhong H, Yang X, Kaplan LM, Molony C, Schadt EE (2010) Integrating pathway analysis and genetics of gene expression for genome-wide association studies. Am J Hum Genet 86: 581–591. 10.1016/j.ajhg.2010.02.020 20346437 PMC2850442

[24] Wang K, Li M, Bucan M (2007) Pathway-Based Approaches for Analysis of Genomewide Association Studies. Am J Hum Genet 81. 10.1086/522374 PMC2276352 17966091

[25] Nicolae DL, Gamazon E, Zhang W, Duan S, Dolan ME, Cox NJ (2010) Trait-Associated SNPs Are More Likely to Be eQTLs: Annotation to Enhance Discovery from GWAS. PLoS Genet 6: e1000888 10.1371/journal.pgen.1000888 20369019 PMC2848547

[26] Segrè AV, Groop L, Mootha VK, Daly MJ, Altshuler D, Consortium D, et al (2010) Common Inherited Variation in Mitochondrial Genes Is Not Enriched for Associations with Type 2 Diabetes or Related Glycemic Traits. PLoS Genet 6: e1001058 10.1371/journal.pgen.1001058 20714348 PMC2920848

[27] Zhang K, Cui S, Chang S, Zhang L, Wang J (2010) i-GSEA4GWAS: a web server for identification of pathways/gene sets associated with traits by applying an improved gene set enrichment analysis to genome-wide association study. Nucleic Acids Research 38: W90–W95. 10.1093/nar/gkq324 20435672 PMC2896119

[28] Wu C, Delano DL, Mitro N, Su SV, Janes J, McClurg P, et al (2008) Gene Set Enrichment in eQTL Data Identifies Novel Annotations and Pathway Regulators. PLoS Genet 4: e1000070 10.1371/journal.pgen.1000070 18464898 PMC2346558

[29] Holmans P, Green EK, Pahwa JS, Ferreira MAR, Purcell SM, Sklar P, et al (2009) Gene Ontology Analysis of GWA Study Data Sets Provides Insights into the Biology of Bipolar Disorder. The American Journal of Human Genetics 85: 13–24. 19539887 10.1016/j.ajhg.2009.05.011 PMC2706963

[30] Kaminski WE, Piehler A, Wenzel JJ (2006) ABC A-subfamily transporters: Structure, function and disease. Biochimica et Biophysica Acta (BBA)—Molecular Basis of Disease 1762: 510–524. 16540294 10.1016/j.bbadis.2006.01.011

[31] Yewdell JW (2005) Immunoproteasomes: regulating the regulator. Proc Natl Acad Sci U S A 102: 9089–9090. 15967978 10.1073/pnas.0504018102 PMC1166640

[32] van Deventer S, Neefjes J (2010) The Immunoproteasome Cleans up after Inflammation. Cell 142: 517–518. 10.1016/j.cell.2010.08.002 20723753

[33] Sijts E, Kloetzel PM (2011) The role of the proteasome in the generation of MHC class I ligands and immune responses. Cellular and Molecular Life Sciences 68: 1491–1502. 10.1007/s00018-011-0657-y 21387144 PMC3071949

[34] Bernassola F, Ciechanover A, Melino G (2010) The ubiquitin proteasome system and its involvement in cell death pathways. Cell Death Differ 17: 1–3. 10.1038/cdd.2009.189 20010850

[35] Wang X, Luo H, Chen H, Duguid W, Wu J (1998) Role of Proteasomes in T Cell Activation and Proliferation. The Journal of Immunology 160: 788–801. 9551914

[36] Witko-Sarsat V, Pederzoli-Ribeil M, Hirsh E, Sozzani S, Cassatella MA (2011) Regulating neutrophil apoptosis: new players enter the game. Trends in immunology 32: 117–124. 10.1016/j.it.2011.01.001 21317039

[37] Shen L, Au W-Y, Guo T, Wong K-Y, Wong ML, Tsuchiyama J, et al (2007) Proteasome inhibitor bortezomib-induced apoptosis in natural killer (NK)–cell leukemia and lymphoma: an in vitro and in vivo preclinical evaluation. Blood 110: 469–470. 17579189 10.1182/blood-2007-02-072900

[38] Yanaba K, Yoshizaki A, Muroi E, Hara T, Ogawa F, Shimizu K, et al (2010) The proteasome inhibitor bortezomib inhibits T cell-dependent inflammatory responses. Journal of Leukocyte Biology 88: 117–122. 10.1189/jlb.1009666 20418448

[39] Okada Y, Hirota T, Kamatani Y, Takahashi A, Ohmiya H, Kumasaka N, et al (2011) Identification of Nine Novel Loci Associated with White Blood Cell Subtypes in a Japanese Population. PLoS Genet 7: e1002067 10.1371/journal.pgen.1002067 21738478 PMC3128095

[40] Reiner AP, Lettre G, Nalls MA, Ganesh SK, Mathias R, Austin MA, et al (2011) Genome-Wide Association Study of White Blood Cell Count in 16,388 African Americans: the Continental Origins and Genetic Epidemiology Network (COGENT). PLoS Genet 7: e1002108 10.1371/journal.pgen.1002108 21738479 PMC3128101

[41] Bellón T, Álvarez L, Mayorga C, Morel E, Torres MJ, Martín-Díaz MA, et al (2010) Differential gene expression in drug hypersensitivity reactions: induction of alarmins in severe bullous diseases. British Journal of Dermatology 162: 1014–1022. 10.1111/j.1365-2133.2009.09627.x 20030638

