# Supplementary figures and images for: ABC Transporters and the Proteasome Complex Are Implicated in Susceptibility to Stevens–Johnson Syndrome and Toxic Epidermal Necrolysis across Multiple Drugs
Source: PLoS One. 2015 Jun 25;10(6):e0131038. doi: 10.1371/journal.pone.0131038 (PMC4482486; doi:10.1371/journal.pone.0131038)

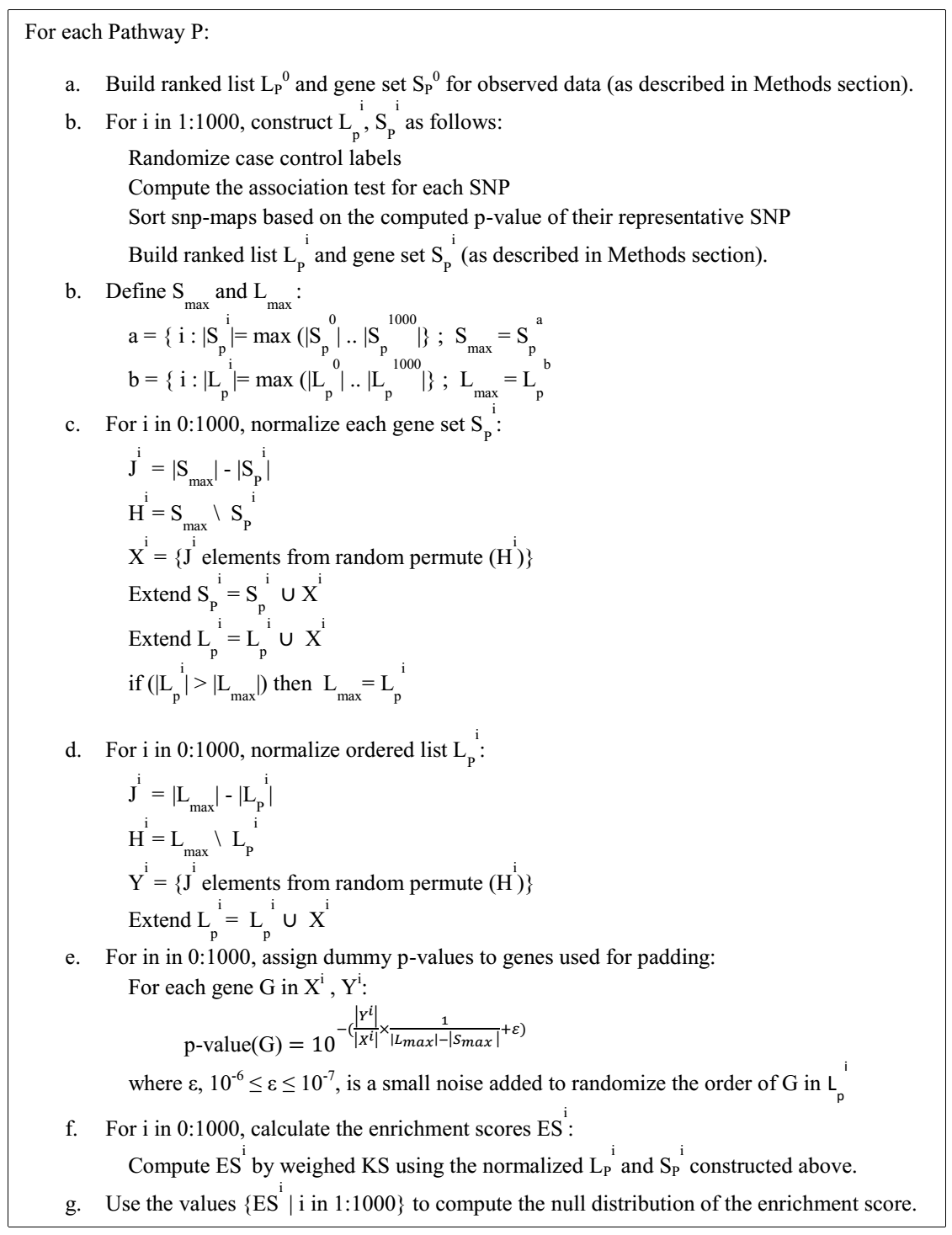

Supplement: S1 Fig — (TIF) [file pone.0131038.s001.tif]

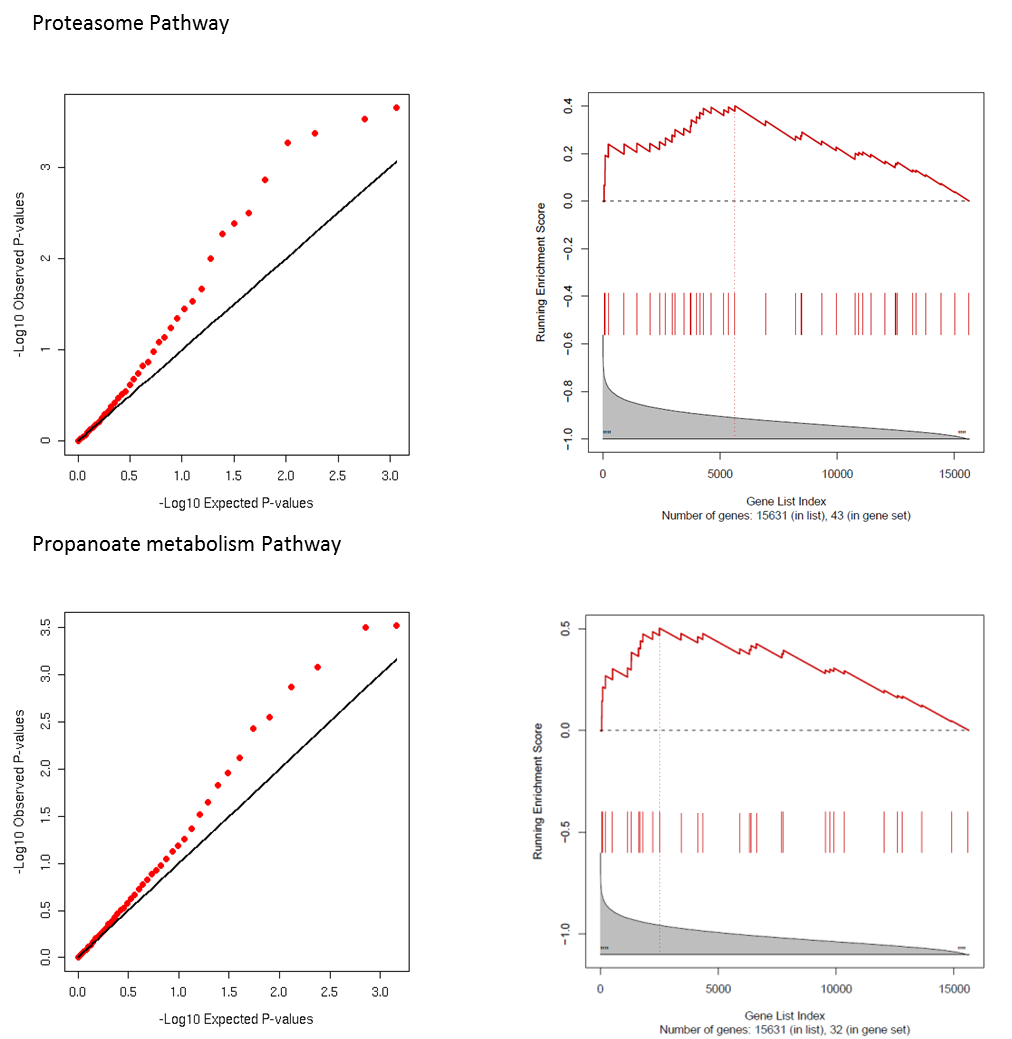

Supplement: S2 Fig — The SNPs presented in each plot are the representative SNPs for all genes in the corresponding pathway. The GSEA plots show the enrichment score of the two pathways. The top portion of each plot shows the running enrichment score for the pathway genes as the analysis moves down the ranked list. The peak score for each plot is the enrichment score for the gene set. The bottom portion of each plot shows the value of the ranking metric as it moves down the list of ranked genes. (TIF) [file pone.0131038.s002.tif]
