# Supplementary material for: ABC Transporters and the Proteasome Complex Are Implicated in Susceptibility to Stevens–Johnson Syndrome and Toxic Epidermal Necrolysis across Multiple Drugs
Source: PLoS One. 2015 Jun 25;10(6):e0131038. doi: 10.1371/journal.pone.0131038 (PMC4482486; doi:10.1371/journal.pone.0131038)
Supplement: S1 File — (DOCX) [file pone.0131038.s003.docx]

**S1 Text: GWAS data availability**

We analyzed previously published GWAS results from 72 Caucasian SJS cases and 461 matched controls [[1](#_ENREF_1)]. Data come from a number of sources including two GlaxoSmithKline studies (PGX40001 [[2](#_ENREF_2)] and LAM30004 [[3](#_ENREF_3)]), the University of Florence, the Population Reference Sample resource (POPRES [[4](#_ENREF_4)]), and the Welcome Trust Case Control Consortium (WTCCC, <http://www.wtccc.org.uk/>). Genotypes for all subjects are available as described in the table below. Data from the LAM30004 study were provided in the context of a collaboration with GSK and have not yet been publicly released. The iSAEC data portal is available at: <https://dataportal.saeconsortium.org/>. The Welcome Trust Case Control Consortium data can be downloaded from the WTCCC web site: <http://www.wtccc.org.uk/>. The identifiers of the samples used are included in the attached Excel spreadsheet (GWAS Sample IDs.xlsx). For data available through the iSAEC data portal we also list the studies where samples come from.

| Source | # cases | # controls | Availability |
| --- | --- | --- | --- |
| PGX40001 | 48 | 66 | International Serious Adverse Events Consortium (iSAEC) |
| LAM30004 | 5 | 31 | From the authors |
| Florence | 19 |  | International Serious Adverse Events Consortium (iSAEC) |
| POPRES |  | 273 | International Serious Adverse Events Consortium (iSAEC) |
| WTCCC |  | 91 | Welcome Trust Case Control Consortium (WTCCC) |

1. Shen, Y., et al., Genome-wide association study of serious blistering skin rash caused by drugs. Pharmacogenomics J, 2011.

2. Pirmohamed, M., et al., Investigation into the multidimensional genetic basis of drug-induced Stevens-Johnson syndrome and toxic epidermal necrolysis. Pharmacogenomics, 2007. 8(12): p. 1661-91.

3. Kazeem, G.R., et al., High-resolution HLA genotyping and severe cutaneous adverse reactions in lamotrigine-treated patients. Pharmacogenet Genomics, 2009. 19 (9): p. 661-5.

4. Nelson, M.R., et al., The Population Reference Sample, POPRES: a resource for population, disease, and pharmacological genetics research. Am J Hum Genet, 2008. 83(3): p. 347-58.
