# Supplementary material for: ABC Transporters and the Proteasome Complex Are Implicated in Susceptibility to Stevens–Johnson Syndrome and Toxic Epidermal Necrolysis across Multiple Drugs
Source: PLoS One. 2015 Jun 25;10(6):e0131038. doi: 10.1371/journal.pone.0131038 (PMC4482486; doi:10.1371/journal.pone.0131038)
Supplement: S2 File — (DOCX) [file pone.0131038.s004.docx]

**S2 Text: Empirical derivation of enrichment score null distribution and Normalized enrichment score**

The null distribution of the enrichment score ES was empirically estimated using 1000 permutations involving random reassignment of case/control labels and re-execution of the GWAS and the GSEA. For each pathway P we compiled the ordered list LPi and the gene set SPi (as described in the main text) for 1000 random runs i, 1 ≤ i ≤ 1000. Due to stochasticity in this step, for any two random runs i and j, the lengths of the lists LPi and LPj as well as genes sets SPi and SPj may be different, which would render enrichment scores from different runs incomparable. To overcome this problem we normalized the size of every SPi and LPi by padding them with extra genes, as described in the pseudo-code of Figure S1. Specifically, SPi is padded with randomly selected pathway genes not already present in SPi; and LPi with randomly selected genes from the entire genome not already present in LPi. To each selected gene we assign a nominal p-value chosen so that the total contribution to ES due to genes added to SPi is the same as the penalty due genes added to LPi. For consistency, the padding procedure is applied not only to the 1000 random runs but also to the original observed data.

Once we obtain comparable sizes for all ordered lists and gene sets, we calculate the null distribution for the enrichment score ES. This distribution is used to compute both the normalized enrichment score (see next section) and the significance of the original ES. The significance of the ES score is calculated as the fraction of the 1,000 random permutations that has greater ES than that observed ES. As noted earlier, this permutation based procedure helps control for biases in the enrichment score due to varying gene and LD-region lengths across gene sets while conserving the gene to gene relationships.

We normalized the enrichment scores to compare them across gene sets of different sizes [[1](#_ENREF_1)]. The normalization is calculated using the method proposed by Wang et al. [[2](#_ENREF_2)]. Specifically, the normalized enrichment score (NES) is calculated by z-score transformation of the enrichment scores, using the mean and standard deviation of enrichment scores of same sign from the null distribution. Finally, we calculate the False Discovery Rate (FDR) for NES using non parametric distribution as proposed in [[1](#_ENREF_1)].

1. Subramanian, A., et al., Gene set enrichment analysis: A knowledge-based approach for interpreting genome-wide expression profiles. Proc Natl Acad Sci U S A, 2005. 102(43): p. 15545-15550.

2. Wang, K., M. Li, and M. Bucan, Pathway-Based Approaches for Analysis of Genomewide Association Studies. Am J Hum Genet, 2007. 81(6).
