# Supplementary material for: ABC Transporters and the Proteasome Complex Are Implicated in Susceptibility to Stevens–Johnson Syndrome and Toxic Epidermal Necrolysis across Multiple Drugs
Source: PLoS One. 2015 Jun 25;10(6):e0131038. doi: 10.1371/journal.pone.0131038 (PMC4482486; doi:10.1371/journal.pone.0131038)
Supplement: S3 File — (DOCX) [file pone.0131038.s005.docx]

**S3 Text: Shadow analysis and Synergy analysis**

To assess the shadow effect, we compared each enriched pathway to all other enriched pathways in a pairwise fashion and in both directions (Path1 vs. Path2 and Path2 vs. Path1). To test if Path1 was a shadow of Path2 we used Pointer to compute the enrichment score (ES) of the set P1 = (Path1 \ Path2), i.e., the genes in Path1 but not in Path2. A null distribution for ES was compiled by computing the enrichment score for 1,000 random subsets of Path1, each of the same size as P1. We considered Path1 to be a shadow of Path2 if (1) the p-value of the ES for P1 was > 0.05, indicating that the genes specific to Path1 are not enriched, and (2) if the p-value of the enrichment score of P2 = (Path2 \ Path1) is <=0.05. Shadow assessment was carried out only for pathway pairs meeting the GSEA minimum size restrictions. To assess synergistic association, all enriched pathways were tested in a pairwise fashion. For a pair of pathways Path1 and Path2, we used Pointer to compute the enrichment score ES of the set of their overlapping genes, O = {Path1 ∩ Path2}, given that O was of size 10 or more. Similar to shadow analysis, a null distribution for ES was compiled by computing the enrichment score for 1,000 sets of genes with the same size as O, randomly selected from the union of the sets Path1 and Path2. We defined as synergistic those pairs where the p-value of ES was ≤ 0.05.
