# Supplementary material for: ABC Transporters and the Proteasome Complex Are Implicated in Susceptibility to Stevens–Johnson Syndrome and Toxic Epidermal Necrolysis across Multiple Drugs
Source: PLoS One. 2015 Jun 25;10(6):e0131038. doi: 10.1371/journal.pone.0131038 (PMC4482486; doi:10.1371/journal.pone.0131038)
Supplement: S1 Table — (DOCX) [file pone.0131038.s006.docx]

**S1 Table: Top associated SNPs to gene in ABC and proteasome pathway.** In the table are reported the top SNPs associated with genes in each of the top two pathways.

| **ABC transporters pathway** | | |  | **Proteasome pathway** | | |
| --- | --- | --- | --- | --- | --- | --- |
| **SNP** | **GENE** | **PV** |  | **SNP** | **GENE** | **PV** |
| rs12040438 | ABCB10 | 0.00011 |  | rs6505294 | PSMD11 | 0.000225 |
| rs640603 | ABCG4 | 0.000332 |  | rs3740049 | PSMD8 | 0.000424 |
| rs4148562 | ABCC5 | 0.000652 |  | rs2072916 | PSMB1 | 0.000491 |
| rs12582525 | ABCA10 | 0.000735 |  | rs1190982 | PSMA3 | 0.001295 |
| rs17758402 | ABCA3 | 0.00083 |  | rs13424110 | PSMD1 | 0.005812 |
| rs497511 | ABCA4 | 0.001077 |  | rs2071481 | PSMB8 | 0.01003 |
| rs4131229 | ABCG5 | 0.001641 |  | rs2071481 | PSMB9 | 0.01003 |
| rs4131229 | ABCG8 | 0.001641 |  | rs10132599 | PSMC1 | 0.01506 |
| rs9516530 | ABCC4 | 0.002248 |  | rs11078936 | PSMD3 | 0.01896 |
| rs5029394 | TAP2 | 0.003156 |  | rs2781553 | PSMA5 | 0.02168 |
| rs7566779 | ABCA12 | 0.003578 |  | rs4346937 | SHFM1 | 0.02477 |
| rs11863504 | ABCC6 | 0.00582 |  | rs546470 | PSMA8 | 0.02624 |
| rs17712293 | ABCA13 | 0.006584 |  | rs647618 | PSMA2 | 0.03044 |
| rs6681849 | ABCD3 | 0.007269 |  | rs6077915 | PSMF1 | 0.03419 |
| rs2235023 | ABCB1 | 0.009284 |  | rs11653487 | PSMB3 | 0.03728 |
| rs2071481 | TAP1 | 0.01003 |  | rs3020900 | PSMD13 | 0.0392 |
| rs2275542 | ABCA1 | 0.01198 |  | rs3742500 | PSME1 | 0.04147 |
| rs7279750 | ABCG1 | 0.01625 |  | rs3742500 | PSME2 | 0.04147 |
| rs4656938 | ABCB5 | 0.01719 |  | rs7125877 | PSMA1 | 0.04601 |
| rs215098 | ABCC1 | 0.01981 |  | rs1330811 | PSMB7 | 0.05453 |
| rs853772 | ABCB11 | 0.02302 |  | rs673604 | PSMB2 | 0.05779 |
| rs2067043 | ABCC8 | 0.02453 |  | rs10500585 | PSMD7 | 0.06302 |
| rs717620 | ABCC2 | 0.02543 |  | rs1877908 | PSME4 | 0.0883 |
| rs10244810 | CFTR | 0.03704 |  | rs234368 | PSMC4 | 0.1183 |
| rs333937 | ABCA5 | 0.04026 |  | rs472054 | PSMA4 | 0.1267 |
| rs4148809 | ABCB4 | 0.04699 |  | rs6432686 | PSMD14 | 0.1526 |
| rs1860447 | ABCA9 | 0.05134 |  | rs11204828 | PSMB4 | 0.1758 |
| rs10150826 | ABCD4 | 0.06071 |  | rs9579214 | POMP | 0.2072 |
| rs1879379 | ABCB9 | 0.07632 |  | rs12536983 | PSMC2 | 0.2077 |
| rs1481017 | ABCG2 | 0.09953 |  | rs17648225 | PSMD12 | 0.2139 |
| rs704205 | ABCC9 | 0.102 |  | rs1219935 | PSME3 | 0.222 |
| rs2153814 | ABCB7 | 0.1054 |  | rs2137144 | PSMC5 | 0.2401 |
| rs17682084 | ABCA8 | 0.1138 |  | rs2069716 | IFNG | 0.2751 |
| rs17822931 | ABCC11 | 0.1334 |  | rs2277459 | PSMA6 | 0.3027 |
| rs12643564 | ABCA2 | 0.1755 |  | rs8010057 | PSMB5 | 0.3044 |
| rs10799 | ABCB6 | 0.1771 |  | rs1892196 | PSMD4 | 0.3113 |
| rs17562467 | ABCC3 | 0.2302 |  | rs11545172 | PSMD2 | 0.3641 |
| rs6947821 | ABCB8 | 0.2326 |  | rs16957378 | PSMB10 | 0.3792 |
| rs13161930 | ABCD2 | 0.2737 |  | rs6142950 | PSMA7 | 0.424 |
| rs7504026 | ABCA6 | 0.3215 |  | rs901746 | PSMC3 | 0.5205 |
| rs17178963 | ABCC10 | 0.451 |  | rs8018404 | PSMC6 | 0.6679 |
| rs3752229 | ABCA7 | 0.4679 |  | rs16833107 | PSMD4 | 0.98 |
| rs7193955 | ABCC12 | 0.5932 |  |  |  |  |
| rs4148030 | ABCD1 | 0.7194 |  |  |  |  |
