# Supplementary material for: ABC Transporters and the Proteasome Complex Are Implicated in Susceptibility to Stevens–Johnson Syndrome and Toxic Epidermal Necrolysis across Multiple Drugs
Source: PLoS One. 2015 Jun 25;10(6):e0131038. doi: 10.1371/journal.pone.0131038 (PMC4482486; doi:10.1371/journal.pone.0131038)
Supplement: S2 Table — (DOCX) [file pone.0131038.s007.docx]

**S2 Table: The two tables show the pathway enrichment results from I-GSEA4GWAS v2 in panel A and from Magenta in panel B**

(A)

| **Gene Set Name** | **P-value** | **FDR value** |
| --- | --- | --- |
| KEGG: TASTE TRANSDUCTION | 0.001 | 0.004 |
| KEGG: GALACTOSE METABOLISM | 0.001 | 0.009 |
| KEGG: GLYCOLYSIS GLUCONEOGENESIS | 0.001 | 0.012 |
| KEGG: PYRUVATE METABOLISM | 0.001 | 0.012 |
| KEGG: BASAL TRANSCRIPTION FACTORS | 0.003 | 0.012 |
| KEGG: INOSITOL PHOSPHATE METABOLISM | 0.001 | 0.012 |
| KEGG: GLYCOSAMINOGLYCAN BIOSYNTHESIS HEPARAN SULFATE | 0.001 | 0.012 |
| KEGG: GLYCOSPHINGOLIPID BIOSYNTHESIS GANGLIO SERIES | 0.001 | 0.012 |
| KEGG: CALCIUM SIGNALING PATHWAY | 0.001 | 0.013 |
| KEGG: PHOSPHATIDYLINOSITOL SIGNALING SYSTEM | 0.001 | 0.016 |
| KEGG: HOMOLOGOUS RECOMBINATION | 0.004 | 0.023 |
| KEGG: ABC TRANSPORTERS | 0.004 | 0.055 |
| KEGG: DILATED CARDIOMYOPATHY | 0.003 | 0.057 |
| KEGG: INTESTINAL IMMUNE NETWORK FOR IGA PRODUCTION | 0.007 | 0.057 |
| KEGG: VASOPRESSIN REGULATED WATER REABSORPTION | 0.012 | 0.083 |

(B)

| **Gene Set Name** | **NOMINAL GSEA PVAL 95 PERC CUTOFF** | **FDR 95 PERC CUTOFF** |
| --- | --- | --- |
| KEGG SYSTEMIC LUPUS ERYTHEMATOSUS | 0.025 | 1 |
| KEGG ABC TRANSPORTERS | 0.036 | 1 |
| KEGG TRYPTOPHAN METABOLISM | 0.036 | 1 |
| KEGG PROTEASOME | 0.057 | 1 |
